# Digital Divide – Soziale Unterschiede in der Nutzung digitaler Gesundheitsangebote

**DOI:** 10.1007/s00103-019-03081-y

**Published:** 2020-01-08

**Authors:** Alejandro Cornejo Müller, Benjamin Wachtler, Thomas Lampert

**Affiliations:** FG28 Soziale Determinanten der Gesundheit, Robert Koch-Institut, General-Pape-Str. 62–66, 12101 Berlin, Deutschland

**Keywords:** Gesundheitliche Ungleichheit, Digitalisierung, Gesundheitskompetenz, Gesundheitliche Chancengleichheit, Digitale Gesundheitsanwendungen, Health inequalities, Digitalisation, Health literacy, Digital healthcare, Health equity

## Abstract

Die gesundheitliche Ungleichheit ist heute eines der wichtigsten Themen für Public Health weltweit. Der Digitalisierung von Gesundheitsangeboten wird dabei häufig das Potenzial zugesprochen, die gesundheitliche Chancengleichheit zu verbessern. Gleichzeitig ist die erfolgreiche Inanspruchnahme von digitalen Gesundheitsangeboten mit der Voraussetzung einer effektiven Nutzung des Internets verbunden, wodurch möglicherweise neue Barrieren für Menschen geschaffen werden, die entweder nicht über die notwendigen materiellen Ressourcen oder die erforderlichen digitalen oder gesundheitlichen Kompetenzen verfügen.

Wie genau sich die Digitalisierung von Gesundheitsangeboten auf die gesundheitliche Chancengleichheit auswirkt, ist bisher wenig erforscht. Ziel dieser Arbeit ist es, einen ersten Überblick über die vorhandene Literatur zu geben. Dabei zeigte ein Großteil der eingeschlossenen Studien, dass die Nutzung von digitalen Gesundheitsangeboten mit soziodemografischen Faktoren assoziiert war. Allgemein war die Inanspruchnahme bei jüngeren Menschen und solchen mit höherer Bildung und höherem Einkommen häufiger. Nur wenige Studien fanden keine Assoziation. Aus anderen Studien ging hervor, dass Menschen mit höherer Gesundheitskompetenz eher digitale Gesundheitsangebote nutzen. Dabei zeigt die Gesundheitskompetenz ebenfalls einen sozialen Gradienten zugunsten jener in höheren sozioökonomischen Positionen. Bei geringer Evidenz gibt es bisher keinen Anhalt für eine Verringerung gesundheitlicher Ungleichheit durch digitale Gesundheitsangebote.

Die analysierten Studien weisen darauf hin, dass bestehende Ungleichheiten sich auch digital fortführen. Es bedarf daher weiterer Forschung, um die Bedeutung von sozialen Determinanten für die digitalen Versorgungsangebote genauer zu verstehen.

## Abstract (en)

Today, health inequalities are one of the most important issues in public health worldwide. The digitalisation of healthcare is frequently attributed with the potential to reduce health inequalities. At the same time, effective internet usage is a prerequisite of the successful utilisation of digital health interventions. This might be a new obstacle for those who lack the necessary material resources or individual skills.

Evidence on how exactly digital health interventions affect health inequalities is scarce. The aim of this study was to present a narrative review of the available literature. The majority of studies showed an association between the usage of digital health interventions and sociodemographic factors. The utilisation was generally higher among younger people and those with higher education and higher income. Only few studies showed no association. Other studies reported higher utilisation among those with higher levels of health literacy while health literacy showed a social gradient to the disadvantage of those in lower socioeconomic position. With a low overall level of evidence, there is currently no indication that digital health interventions are reducing health inequalities.

The studies analysed in this review indicate that existing inequalities persist in the digital realm. Further evidence is needed to create a better understanding of the importance of sociodemographic factors for digital health interventions.

## Einleitung

Die Digitalisierung im Gesundheitswesen kann für die Prävention von Krankheiten und die Versorgung der Bevölkerung große Chancen bieten. Dieser positive Effekt der Digitalisierung ist allerdings an einige Voraussetzungen gebunden. Digitale Versorgungsformen oder Präventionsangebote dürfen keine Scheininnovationen sein, sondern müssen ihren Nutzen für die Versorgung nachweisen. Sie müssen sowohl medizinisch sicher sein als auch konform zum Datenschutz. Darüber hinaus sollten digitale Gesundheitsangebote für alle Anwender verständlich sein, sodass möglichst alle Menschen an diesem medizinischen Fortschritt teilhaben [1].

Soziale Determinanten wie Bildung, Berufsstatus und Einkommen haben beträchtliche Auswirkungen auf die Gesundheit. Ein niedriger sozialer Status geht mit einer niedrigeren Lebenserwartung, höheren Risiken für chronische Erkrankungen und einem höheren Risiko für Berufsunfähigkeit einher [2]. Da sich bei sozial Benachteiligten von Geburt an größere Gesundheitsbelastungen summieren, unter anderem infolge von ungünstigeren Lebensbedingungen und riskanterem Gesundheitsverhalten, spricht man in diesem Zusammenhang auch von schlechteren Gesundheitschancen bzw. gesundheitlicher Chancenungleichheit [3].

Digitalen Gesundheitsangeboten wurde schon früh das Potenzial zugesprochen, die gesundheitliche Chancengleichheit zu verbessern [4]. Niedrigschwellige Barrieren, schnelle Implementierung, Personalisierbarkeit und einfache Skalierungseffekte sprechen für dieses Potenzial. Von einer Digitalisierung von Versorgungsformen und digitalen Präventionsangeboten kann jedoch nur profitieren, wer auch tatsächlich Zugang zum Internet hat und über entsprechende Hardware verfügt. Zugang und Hardware sind jedoch ungleich verteilt, man spricht in diesem Kontext auch vom „Digital Divide“ [5]. Mit der steigenden Verfügbarkeit von Internetzugang zeigt sich der Digital Divide zunehmend in Ungleichheiten von digitalen Kompetenzen und Unterschieden im Nutzungsverhalten [6, 7]. Im Kontext der digitalen Gesundheitsangebote ist dies von hoher Relevanz, da diese, je nach Ausgestaltung, eine größere Einbindung der Nutzerinnen und Nutzer sowie Eigenverantwortung voraussetzen [8, 9]. Die individuelle Gesundheitskompetenz sowie die digitale Kompetenz des Einzelnen kann also einen starken Einfluss darauf haben, wie gut digitale Versorgungs- und Präventionsangebote in Anspruch genommen werden und wie effektiv diese Angebote genutzt werden.

Aufgrund des Digital Divide ist anzunehmen, dass jene Bevölkerungsgruppen, die heute schon die größte Krankheitslast tragen, von digitalen Versorgungsformen und Präventionsangeboten am wenigsten profitieren und sich dadurch die gesundheitliche Chancenungleichheit weiter vergrößert [9–11]. Um diese These zu prüfen, werden im Folgenden die Ergebnisse von Studien berichtet, die sich mit dieser Frage befasst haben. Da für Deutschland bislang nur wenige Studien vorliegen, werden zum Teil auch Ergebnisse aus Studien anderer Länder dargestellt. Insgesamt wurden in diese explorative Analyse 16 Studien eingeschlossen, die sich bezüglich des Designs, der Methodik und der Studienpopulation stark voneinander unterscheiden. Dies ist bei der Interpretation und Einordnung der Ergebnisse zu berücksichtigen. Der Schwerpunkt liegt im Folgenden auf Studien, die soziale Unterschiede in der Nutzung von digitalen Angeboten der Prävention und Gesundheitsversorgung untersucht haben. Ergänzend dazu werden Ergebnisse von Studien einbezogen, die soziale Unterschiede in Bezug auf die Kompetenz zur Nutzung digitaler Gesundheitsangebote betrachtet haben (E-Health Literacy). Vorab wird kurz auf das Konzept und die Definition von Digital Divide eingegangen.

## Konzept und Definition von Digital Divide

Schon heute haben zahlreiche systematische Reviews gezeigt, dass digitale Interventionen, besonders im präventiven Bereich, effektiv sein können [12–16]. Die meisten digitalen Gesundheitsangebote zielen auf individuelle Verhaltensänderungen bei den Anwenderinnen und Anwendern ab und übertragen ihnen damit viel (Eigen‑)Verantwortung. Der Begriff *Digital Divide* stellt darauf ab, dass Menschen mit niedrigem sozialen Status weniger stark am digitalen Wandel partizipieren und so auch von neuen digitalen Versorgungsformen möglicherweise weniger profitieren [9]. Dabei wird heute zwischen drei Formen des Digital Divide unterschieden [7]: Der primäre Digital Divide beschreibt den mangelnden Zugang zu Technologie und Internet. Dieser verliert jedoch mit der rasanten Verbreitung von Internet und Technologie zunehmend an Bedeutung [17]. Umso wichtiger werden dadurch der sekundäre und tertiäre Digital Divide. Der sekundäre beschreibt Disparitäten in Bezug auf Nutzungsmuster und die Fähigkeiten, digitale Technologien zu bedienen oder im Internet gezielt zu navigieren [7]. Der tertiäre Digital Divide bezeichnet Unterschiede in der Fähigkeit, mithilfe von digitalen Technologien ein verbessertes (gesundheitliches) Outcome zu erzielen [18].

Die Ebenen des Digital Divide sind sukzessive Hürden bei der Inanspruchnahme von digitalen Gesundheitsangeboten. Aufgrund dieser Entwicklung ist es für Politik, Krankenkassen, Entwickler und Leistungserbringer wichtig zu verstehen, wie digitale Gesundheitsangebote in Abhängigkeit von sozialen, ökonomischen und demografischen Faktoren akzeptiert und (effektiv) genutzt werden. Denn es besteht die Gefahr, dass sich bestehende soziale und gesundheitliche Ungleichheiten in digitalen Ungleichheiten fortsetzen und so effektive Versorgungsangebote jene nicht erreichen, die sie am dringendsten benötigen [9, 11].

## Inanspruchnahme von digitalen Präventionsangeboten

Ein großer Anteil der digitalen Gesundheitsangebote ist darauf ausgelegt, gesundheitsförderliche oder präventive Wirkungen zu erzielen, indem eine nachhaltige Verbesserung des Lebensstils adressiert wird. So fand die CHARISMHA(Chances and Risks of Mobile Health Apps)-Studie im Bereich Prävention die meisten Angebote [19]. Durch die digitale Unterstützung von gesundheitsfördernden Verhaltensänderungen, bspw. körperliche Aktivität oder gesünderes Essen, sollen Risikofaktoren wie etwa Übergewicht wirksam reduziert werden, bevor das Gesundheitssystem in Anspruch genommen wird. Erste Evidenz zeigt auch, dass solche Gesundheitsangebote effektiv sein können. Dennoch erfordern sie, anders als bei der Verhältnisprävention, Kenntnis, Engagement und Verantwortung der Einzelnen. Aufgrund des Digital Divide und weiterer sozialer Einflussfaktoren profitieren verschiedene Teile der Bevölkerung daher möglicherweise unterschiedlich stark.

Ein Schwerpunkt von digitalen Gesundheitsförderungs- und Präventionsangeboten liegt im Bereich der mobilen Anwendungen (M-Health). Damit sind besonders Softwareapplikationen (Apps) gemeint, die auf mobilen Endgeräten, meist dem Smartphone, installiert werden und einen Gesundheitsbezug haben. Dabei reicht das Spektrum von Apps, die allgemeine Gesundheitsinformationen oder Ernährungsinformationen bereitstellen, über Apps zur Motivation zu Verhaltensänderungen bis hin zu Apps mit Erinnerungsfunktion für etablierte medizinische Präventionsangebote, wie z. B. Impftermine. In den App-Stores der beiden großen Anbieter von Betriebssystemen mobiler Endgeräte befanden sich 2015 plattformübergreifend bereits ca. 100.000 Apps, die dem Bereich Gesundheit zugeordnet werden konnten [19]. Es ist anzunehmen, dass diese Zahl seitdem deutlich zugenommen hat. Der überwiegende Teil dieser Angebote ist für die Nutzenden kostenlos und wird durch die Privatwirtschaft zur Verfügung gestellt.

Eine Marktforschungsstudie durch die Bitkom Research GmbH von 2017 zeigte, dass 45 % aller deutschen Smartphonebesitzerinnen und -besitzer über 14 Jahre mindestens eine Gesundheits-App nutzten [20]. Dabei waren solche Apps am beliebtesten, die Daten zur körperlichen Funktion (Blutdruck, Herzfrequenz) oder Fitnessdaten, wie z. B. die Anzahl der gegangenen Schritte, registrieren. 27 % der Befragten gaben an, solche Apps zu nutzen. An zweiter Stelle wurden Apps genannt, die über Gesundheits- und Ernährungsthemen informieren (20 %), vor Apps, die aufgrund von aufgezeichneten Gesundheitsdaten der Nutzenden Motivations- und Verhaltensratschläge bereitstellen (11 %), und Apps, die an medizinische Interventionen wie Medikamenteneinnahme oder Impfungen erinnern sollen (2 %). Eine wiederholte Marktforschungsbefragung des gleichen Anbieters im Jahr 2019 zeigte, dass bereits zwei von drei über 16-jährigen deutschen Smartphonebesitzenden mindestens eine Gesundheits-App nutzten. Dabei waren die Informations-Apps die beliebtesten Anwendungen (25 %) vor sogenannten Tracking-Apps (24 %) und Work-out-Apps (17 %) [21]. Eine Studie im Auftrag der Techniker Krankenkasse zeigte, dass die Nutzung von Gesundheits-Apps insgesamt vom Einkommen abhängig war. Dabei war der Anteil der Nutzerinnen und Nutzer solcher Angebote in der höchsten Einkommensgruppe ungefähr doppelt so hoch wie in der niedrigsten. Befragt wurden 1002 Erwachsene zwischen 18 und 70 Jahren [22].

Internationale Studien zeigen ebenfalls, dass die Nutzung von Gesundheits-Apps auf mobilen Endgeräten in der Gesellschaft ungleich verteilt ist. Eine Studie aus den USA zeigte, dass insgesamt 22 % der befragten Erwachsenen in der HINTS(Health Information National Trends Survey)-Studie im Jahr 2014 mindestens eine Gesundheits-App nutzten [23]. Dabei hatten Menschen mit einer akademischen Ausbildung gegenüber denen ohne Highschoolabschluss eine 2,8-fach erhöhte Chance, eine Gesundheits-App zu nutzen. Eine Auswertung der Daten aus der HINTS-Studie zeigte ebenfalls, dass höhere Bildung mit häufigerer Nutzung von digitalen Gesundheitsangeboten assoziiert war [24]. In derselben Studie konnte auch eine Assoziation zwischen den Faktoren Einkommen und Arbeitslosigkeit und der Nutzung von Gesundheitsangeboten festgestellt werden.

Auch eine Studie aus den Niederlanden weist auf soziale Unterschiede in der Nutzung von Gesundheits-Apps hin [25]. Insgesamt gaben 28,7 % der erwachsenen Befragten eines repräsentativen Samples an, mindestens eine Gesundheits-App zu nutzen. Dabei waren die Nutzenden im Vergleich zu Nichtnutzenden jünger und höher gebildet. Auch zeigten sie eine höhere E‑Health Literacy. Die Autorinnen konnten zudem zeigen, dass Unterschiede in der Nutzung von Gesundheits-Apps zwischen den verschiedenen App-Kategorien auftreten. So nutzten Männer Fitness-Apps häufiger als Frauen, wohingegen Frauen häufiger Ernährungs- und Selbstfürsorge-Apps sowie Apps zur reproduktiven Gesundheit nutzten. Bildung korrelierte besonders stark mit der Nutzung von Achtsamkeits-Apps, mit einer höheren Chance der Nutzung für jene mit höherer Bildung.

Eine Korrelation zwischen häufigerer Nutzung von Gesundheits-Apps und höherem Bildungsgrad konnte auch in einer Befragung von 904 Erwachsenen in den USA festgestellt werden. Die Chance, dass eine Person mit Universitätsabschluss eine Gesundheits-App heruntergeladen hatte, war bei der Befragung etwa 3‑mal höher (OR 2,93, 95 % CI 1,43–6,00) im Vergleich zu Personen mit Highschoolabschluss oder geringerem Bildungsgrad. Zudem zeigte die Studie, dass bei Befragten, die in den USA zu den Minderheiten zählen, darunter Lateinamerikaner und Menschen koreanischer Herkunft, die Chance, eine App heruntergeladen zu haben, deutlich verringert war [26].

Neben Apps sind Mobilgeräte (Mobile Devices), die ein Monitoring verschiedener Körperfunktionen ermöglichen (Wearables, Smartwatches, Fitnesstracker etc.), eine weitere Möglichkeit, Prävention digital zu unterstützen. Mobile Devices sind über die letzten Jahre einer der größten Fitnesstrends international [27]. Markt- und Meinungsforschungsuntersuchungen zeigten, dass in Deutschland 31 % der über 14-Jährigen eine Form des Fitnesstrackings nutzten [28]. Laut einer anderen Umfrage nutzten 15 % der Frauen und 17 % der Männer im Jahr 2017 eine Smartwatch oder einen Fitnesstracker am Handgelenk [29].

In einer französischen Studie wurden die Teilnehmenden mit Fitnesstrackern ausgestattet und die Dauer der Nutzung ermittelt. Nach sechs Monaten trugen noch etwa 50 % der Teilnehmenden den Tracker, nach 300 Tagen waren es nur noch 12 %. Der Beobachtungszeitraum betrug 320 Tage. Assoziiert mit einer längeren Nutzung der Tracker war die subjektiv empfundene Nützlichkeit des Trackers. Ein Einfluss von sozioökonomischen Faktoren konnte nicht festgestellt werden [30].

In einer Studie in den USA mit 4974 Teilnehmenden zeigte sich, dass eine niedrigere Gesundheitskompetenz mit einer geringeren Nutzung von Aktivitätstrackern assoziiert war. Eine höhere Gesundheitskompetenz war assoziiert mit einer höheren subjektiv empfundenen Benutzerfreundlichkeit und Nützlichkeit. Darüber hinaus konnte in dieser Studie auch festgestellt werden, dass eine niedrigere Gesundheitskompetenz negativ mit der Häufigkeit der Nutzung von anderen digitalen Gesundheitsangeboten wie Fitness-Apps, Ernährungs-Apps oder Patientenportalen korreliert [31].

## Inanspruchnahme von digitalen Versorgungsangeboten

Digitale Versorgungsangebote werden zunehmend Einzug in den Versorgungsalltag halten. Das durch den Bundestag beschlossene „Digitale Versorgungsgesetz“ (DVG) ist auch Ausdruck des politischen Willens, digitale Versorgungsformen stärker in den Behandlungsalltag zu integrieren, sowohl im Rahmen der Prävention als auch im Rahmen der Diagnose, Therapie und des Monitorings bzw. Krankheitsmanagements. Dabei setzen viele der digitalen Versorgungsformen auf ein stärkeres Selbstmanagement der Patientinnen und Patienten und übertragen diesen mehr Eigenverantwortung für ihre Gesundheit. Auf diesen Umstand hat schon der Sachverständigenrat für Verbraucherfragen im Jahr 2016 hingewiesen [8]. Auch in der Begründung des DVG heißt es, dass viele medizinische Versorgungsangebote zunehmend auf das Selbstmanagement und das Informationsbedürfnis von Patientinnen und Patienten setzen. Daher sollen auch Krankenkassen im Rahmen von Kooperationen mit Herstellern und Anbietern digitaler Medizinprodukte die Möglichkeit erhalten, neue Wege in der Versorgung umzusetzen [32]. Wie gut diese neuen Versorgungsformen von verschiedenen Bevölkerungsgruppen angenommen werden, ist jedoch noch wenig untersucht.

Eine niederländische Studie untersuchte das Interesse von Diabeteserkrankten an der Nutzung einer Telemonitoringplattform. Dabei wurden Unterschiede in der Bereitschaft, eine solche Plattform zu nutzen, festgestellt. Eine höhere Bereitschaft zur Nutzung gab es insbesondere bei jüngeren, besser ausgebildeten und mit dem Internet vertrauten Teilnehmerinnen und Teilnehmern [33].

Die Präferenz gegenüber analogen, persönlichen Versorgungsangeboten (Face to Face oder per Telefon) wurde in einem großen US-amerikanischen Versorgungsnetzwerk erfragt. In der Studie von Graetz et al. gaben mehr als zwei Drittel der Teilnehmenden an, einen persönlichen Kontakt vorzuziehen. Dabei konnte aber kein signifikanter Einfluss von Bildung, Einkommen oder Alter auf die Präferenz von Onlinekommunikation gegenüber persönlichem Kontakt festgestellt werden. In einem zweiten Teil der Studie wurde die Nutzung des Patientenportals des Versorgungsnetzwerkes erfragt. Dabei zeigte sich, dass Befragte mit geringerer Bildung dieses seltener nutzen [34].

Gordon et al. analysierten ebenfalls Daten und Befragungen aus einem großen US-amerikanischen Versorgungsnetzwerk (Kaiser Permanente). Dabei flossen die Daten von Versicherten im Alter von 65 bis 79 Jahren ein. Es zeigte sich, dass die älteren Versicherten von 70 bis 74 und 75 bis 79 Jahren signifikant seltener für den Zugang in das untersuchte Patientenportal registriert waren und, wenn sie registriert waren, sie die einzelnen Funktionen wie „Nachrichten an einen Behandler senden“, „Laborberichte ansehen“ und „Medikamente nachbestellen“ weniger nutzten. Bei der Analyse konnten nicht nur Unterschiede nach Alter, sondern auch nach Ethnie und Bildungsgrad festgestellt werden [35].

In einer deutschen Studie wurden 374 stationär aufgenommene Patientinnen und Patienten zu einem webbasierten Nachsorgeprogramm befragt und ihre Bereitschaft untersucht, ein solches Angebot zu nutzen. Dabei war die Bereitschaft insgesamt niedrig. Die höchste Bereitschaft konnte unter Jugendlichen und jungen Erwachsenen im Alter von 14 bis 26 Jahren festgestellt werden. Weiter konnten signifikante Assoziationen zwischen höherer Akzeptanz und vorheriger E‑Health-Nutzung, höherem Bildungsgrad und privatem Internetanschluss identifiziert werden. Als weitere signifikante Faktoren für die Akzeptanz einer solchen Versorgung konnten in der Analyse das soziale Umfeld, der erwartete Aufwand und der erwartete Nutzen identifiziert werden [36].

Casillas et al. betrachteten den Zusammenhang zwischen der Nutzung der Medikamentenbestellung über ein Patientenportal und der Medikationsadhärenz als Outcome in einer Querschnittstudie. Die Nutzung des Patientenportals zur Nachbestellung von (zuvor verschriebenen) Medikamenten ging mit einer höheren Adhärenz bei der Einnahme der Medikamente einher. Die Studie untersuchte vorrangig Unterschiede zwischen Teilnehmenden, die Englisch sprachen, und solchen, deren Englischkenntnisse eingeschränkt waren. Ein Vergleich unter den englischsprachigen Teilnehmenden zeigte, dass höhere Bildung, höheres Einkommen und jüngeres Alter prädiktiv für häufigere Bestellung von Medikamenten waren. Diese Variablen waren jedoch nicht prädiktiv bei Teilnehmenden mit einer anderen Muttersprache als Englisch. Die Studie weist auch darauf hin, dass eine Differenz bei der Inanspruchnahme der Nachbestellung zwischen den untersuchten Gruppen, englischsprachig und nichtenglischsprachig, besteht. Dabei wurde das Angebot von Letzteren seltener in Anspruch genommen und auch die Medikationsadhärenz war in dieser Gruppe schlechter [37].

## E-Health Literacy

Die zunehmende Digitalisierung von traditionellen Angeboten des Gesundheitssystems (z. B. durch Onlineterminvereinbarungen, digitale Rezepte) und die große Menge unkontrollierter und aus unterschiedlichsten Quellen stammender digitaler Gesundheitsinformationen und -angebote können Patientinnen und Patienten heute vor größere Herausforderungen stellen. Es gilt, eine große Menge unterschiedlicher digitaler Informationen auf ihre gesundheitliche Relevanz zu überprüfen, einzuordnen und sie so einzusetzen, dass sie zur Verbesserung der eigenen Gesundheit führen. *Health Literacy *(Gesundheitskompetenz) ist ein Begriff, der in den 1970er-Jahren in die Gesundheitswissenschaften eingeführte wurde [38]. Er bezieht sich in seiner ursprünglichen Form auf individuelle Kompetenzen und Ressourcen, die es Menschen ermöglichen, Gesundheitsinformationen zur Aufrechterhaltung und Förderung der eigenen Gesundheit zu nutzen [39]. Das Konzept der Health Literacy ist seitdem stetig weiterentwickelt worden und es existiert heute eine Vielzahl unterschiedlicher Definitionen [40], die verschiedene Teilaspekte von Health Literacy betonen. Sørensen et al. fassen ihre Analyse von 17 verschiedenen Definitionen folgendermaßen zusammen: „Health literacy is linked to literacy and entails people’s knowledge, motivation and competence to access, understand, appraise, and apply health information in order to make judgements and take decisions in everyday life concerning healthcare, disease prevention and health promotion to maintain or improve quality of life during the life course“ [40].

Health Literacy ist heute eines der wichtigsten Konzepte im Bereich der Gesundheitsförderung, wie die Erklärung am Ende der 9. Globalen Konferenz zur Gesundheitsförderung der Weltgesundheitsorganisation (WHO) in Shanghai zeigte [41]. Während in der Shanghai-Deklaration die Potenziale von Health Literacy für die Nutzbarmachung der digitalen Technologien zur Gesundheitsförderung und die Verbesserung gesundheitlicher Chancengleichheit betont werden, sehen andere Arbeiten die Gefahr einer möglichen Vergrößerung von gesundheitlicher Ungleichheit durch eine ungleich in der Gesellschaft verteilte *E‑Health Literacy *[42], die als die Fähigkeit definiert wird, neue Informationen aus elektronischen Ressourcen zu suchen, zu verstehen und einzuschätzen und das daraus gewonnene Wissen zur Lösung von Gesundheitsproblemen anzuwenden [43]. Wenige Studien haben bisher die Bedeutung von Health Literacy und E‑Health Literacy für die gesundheitliche Ungleichheit untersucht [44, 45]. Eine aktuelle Studie aus den USA zeigte, dass Menschen mit einem hohen Risiko für gesundheitliche Benachteiligungen, also jene mit niedrigem Einkommen, niedriger Bildung und Zugehörigkeit zu einer ethnischen Minderheit, gleichzeitig auch die niedrigsten Level an Gesundheitskompetenz hatten [46]. Bennett et al. zeigten in einer ebenfalls in den USA durchgeführten Studie, dass Health Literacy dabei ein wichtiger Mediator ist, der Unterschiede in subjektiver Gesundheit nach Bildung und ethnischer Zugehörigkeit zum Teil erklären kann [47], ein Ergebnis, das auch durch eine systematische Übersicht bestätigt wurde [45]. Die ungleiche Verteilung von Health Literacy zwischen verschiedenen sozioökonomischen Gruppen ist dabei übereinstimmend in unterschiedlichen Populationen und zu unterschiedlichen Zeitpunkten nachgewiesen worden [48, 49], wobei Menschen mit geringer Bildung oder geringem Einkommen, geringen Kenntnissen der Landessprache und Zugehörigkeit zu ethnischen Minderheiten mit höherer Wahrscheinlichkeit eine niedrige Gesundheitskompetenz hatten. Ähnliche erste Ergebnisse liegen auch für Deutschland vor. Auch hier zeigte sich, dass soziodemografische Faktoren, wie ein höheres Lebensalter, ein niedriges Bildungsniveau, das Vorhandensein eines Migrationshintergrunds und ein allgemein niedriger Sozialstatus, mit einer eingeschränkten Gesundheitskompetenz assoziiert sind [50, 51]. Auch spezifisch für E‑Health Literacy wurden soziodemografische Unterschiede beschrieben, wobei v. a. ältere Menschen und solche mit geringerer Bildung konsistent eine geringere E‑Health Literacy zeigten [42, 52]. Inwieweit dieser Umstand zu den ebenfalls in diesen Gruppen erhöhten schlechteren Gesundheitsoutcomes beiträgt, ist bisher nur unzureichend erforscht [53].

Die beschriebenen Zusammenhänge zwischen soziodemografischen Eigenschaften und Gesundheitskompetenz und der Assoziation zwischen dieser und diversen Gesundheitsoutcomes lassen es plausibel erscheinen, dass eine zunehmende Digitalisierung ohne gezielte Förderung von Gesundheitskompetenzen der Benutzerinnen und Benutzer und ohne spezifische Ausrichtung an den Bedürfnissen derjenigen mit geringer digitaler Nutzungskompetenz die soziale Ungleichheit in der Gesundheit vergrößern kann. Neue digitale Gesundheitsdienstleistungen und Informationen, die immer komplexere Fähigkeiten und Kompetenzen für die erfolgreiche Nutzung und Integration benötigen, werden wahrscheinlich v. a. Menschen zugutekommen, die bereits privilegierten Zugang zu gesundheitsförderlichen Ressourcen haben. Wenn diese neuen digitalen Interventionen einen positiven Gesundheitseffekt aufweisen, können sich somit gesundheitliche Ungleichheiten vergrößern. Gleichzeitig wird Health Literacy immer wieder als Instrument benannt, durch welches sich gesundheitliche Ungleichheiten verringern ließen, vor allem mit Bezug auf die Potenziale digitaler Technologien [41, 44, 54]. Dabei ist es wichtig zu beachten, dass die Gesundheitskompetenz, die in den hier genannten Studien untersucht wurde, meist auf die individuellen Fähigkeiten ausgerichtet ist. Gleichzeitig hat sich Gesundheitskompetenz als Konzept mittlerweile deutlich weiterentwickelt und bezieht sich heute zunehmend auf die sozialen Ressourcen, die Gesundheitskompetenz ermöglichen. Für Gesundheitssysteme und Gesundheitsförderung stellt sich die Frage, wie sie sich organisieren sollten, um soziale Ressourcen zu stärken und damit Gesundheitskompetenz zu ermöglichen und zu fördern [55, 56]. Diese Anforderungen an die Gesundheitssysteme werden mit den zunehmend voraussetzungsreichen und komplexen digitalen Gesundheitsangeboten wachsen, wenn die gesundheitliche Ungleichheit gleichzeitig vermindert werden soll.

## Fazit

Die analysierten Studien zeigen in weitgehender Übereinstimmung, dass zum Teil beträchtliche soziale Unterschiede in der Nutzung von digitalen Angeboten bestehen. Dies gilt sowohl hinsichtlich digitaler Angebote der Prävention und Gesundheitsförderung als auch für digitale Angebote der Versorgung. Die sozialen Unterschiede sind nicht allein auf Unterschiede im Zugang zum Internet und in der Verfügbarkeit entsprechender Hardware zurückzuführen, sondern auch und insbesondere auf Unterschiede in Bezug auf E‑Health Literacy. Allerdings gibt es bislang nur wenige Studien, die sich mit dem Thema Digital Divide befasst haben. Das trifft besonders auf Deutschland zu, aber auch international sind aussagekräftige Studien noch selten. Bei insgesamt geringer Evidenz gibt es bisher auch keinen Anhalt für eine Verringerung gesundheitlicher Ungleichheit durch digitale Gesundheitsangebote. Die Nutzungsmuster in den analysierten Studien weisen viel mehr darauf hin, dass bestehende Ungleichheiten sich auch digital fortführen oder gar vergrößern. Es bedarf daher weiterer Forschung, um die Bedeutung von sozialen Determinanten für die Akzeptanz und Effektivität digitaler Versorgungsangebote und die Kausalzusammenhänge genauer zu verstehen.

So könnten unterschiedliche soziodemografische Faktoren, wie das Alter oder der soziale Status, die Wahrscheinlichkeit der Nutzung digitaler Angebote direkt beeinflussen. Denkbar ist auch, dass die Gesundheitskompetenz als ein möglicher Mediator zwischen dem sozioökonomischen Status und der Wahrscheinlichkeit der Nutzung digitaler Angebote agiert. Diese sozioökonomischen und -demografischen Faktoren könnten möglicherweise auch selbst Mediatoren der Wirkung von (digitalen) Gesundheitsangeboten in unterschiedlichen sozioökonomischen Gruppen sein. Diese Zusammenhänge gilt es, besser zu verstehen.

Zur Untersuchung der Akzeptanz von digitalen Gesundheitsangeboten sind insbesondere qualitative Studien geeignet. Diese könnten Motivationen und Barrieren sehr gut offenlegen. Die Nutzung von digitalen Gesundheitsangeboten sollte in repräsentativen Studien abgefragt werden, um eine Generalisierbarkeit von Ergebnissen zu gewährleisten. Gleichzeitig kann über longitudinale Studien auch die Entwicklung der Inanspruchnahme digitaler Angebote untersucht werden. Nur so wird es möglich, langfristige Trends abzuleiten, zu erkennen und möglichen Fehlentwicklungen in Bezug auf die gesundheitliche Ungleichheit entgegenzuwirken. Letztlich müssen die richtigen Methoden gefunden werden, um die Effektivität von digitalen Gesundheitsangeboten für unterschiedliche soziodemografische Gruppen zu evaluieren. Dabei sollte die Evaluation von digitalen Interventionen immer die sozialen Ungleichheiten und individuellen Voraussetzungen mitberücksichtigen. So kann auch ein tieferes Verständnis darüber erreicht werden, wie die verschiedenen soziodemografischen Einflussfaktoren im Zusammenhang mit digitalen Gesundheitsangeboten wirken und welche Faktoren besonders relevant sind. Bildung scheint nach den bisherigen Untersuchungen einen besonders starken Einfluss zu haben. Darüber hinaus könnten auch kumulative Effekte und Interaktionen verschiedener Einflussfaktoren besser untersucht werden und damit könnte letztlich mehr über die kausalen Zusammenhänge gesagt werden.

Nur wenn die in der Gesellschaft ungleichen Voraussetzungen in Bezug auf Gesundheitskompetenz und digitale Kompetenz auch bei der Entwicklung der digitalen Angebote schon mitgedacht werden, kann die Digitalisierung von Gesundheitsangeboten ihr Nutzenversprechen, zugänglichere und kostengünstigere Versorgung zu gewährleisten, realisieren. Werden die sozialen und individuellen Voraussetzungen nicht berücksichtigt, werden insbesondere solche Patientinnen und Patienten ausgeschlossen, die heute schon die höchste Krankheitslast tragen. Entwickler und Industrie sollten daher dafür Sorge tragen, dass digitale Gesundheitsangebote so ausgestaltet sind, dass sie für alle Endnutzergruppen eine adäquate Nutzerfreundlichkeit aufweisen. Usability von digitalen Produkten wird damit zu einem Merkmal für effektive digitale Gesundheitsangebote. Entsprechend sollte sie auch über alle Nutzergruppen hinweg konsequent evaluiert werden. Darüber hinaus sollten Entwickler und Unternehmen dafür Sorge tragen, dass digitale Angebote auf vielen verschiedenen Endgeräten laufen, um möglichst allen potenziellen Nutzerinnen und Nutzern den Zugang zu ermöglichen.

Für bereits bestehende effektive digitale Gesundheitsangebote, die einen realen Zusatznutzen aufweisen, sind passende Rahmenbedingungen sicherzustellen, um sie in die Regelversorgung übernehmen zu können. Daraus folgt auch, dass digitale Versorgungsangebote ihre Effektivität nachweisen müssen. Dafür müssen verlässliche Instrumente geschaffen werden, die den speziellen Anforderungen von digitalen Gesundheitsangeboten gerecht werden. Letztlich muss aber auch in Zukunft gewährleistet bleiben, dass es für Menschen, die keine digitalen Angebote nutzen, zugängliche Alternativen gibt.
