# Erratum zu: Digital Divide – Soziale Unterschiede in der Nutzung digitaler Gesundheitsangebote

**DOI:** 10.1007/s00103-021-03326-9

**Published:** 2021-05-06

**Authors:** Alejandro Cornejo Müller, Benjamin Wachtler, Thomas Lampert

**Affiliations:** FG28 Soziale Determinanten der Gesundheit, Robert Koch-Institut, General-Pape-Str. 62–66, 12101 Berlin, Deutschland

**Erratum zu:**

**Bundesgesundheitsbl 2019**

10.1007/s00103-019-03081-y

Der Artikel Digital Divide – Soziale Unterschiede in der Nutzung digitaler Gesundheitsangebote von Alejandro Cornejo Müller, Benjamin Wachtler und Thomas Lampert wurde ursprünglich am 08.01.2020 ohne Open Access online auf der Internetplattform des Verlags publiziert. Die Autoren haben sich jedoch nachträglich für eine Open-Access-Veröffentlichung entschieden. Das Urheberrecht des Artikels wurde deshalb am 29.03.2021 in © The Author(s) 2020 geändert. Der Artikel wird nun unter der Creative-Commons-Namensnennung 4.0 International Lizenz veröffentlicht, welche die Nutzung, Vervielfältigung, Bearbeitung, Verbreitung und Wiedergabe in jeglichem Medium und Format erlaubt, sofern Sie den/die ursprünglichen Autor(en) und die Quelle ordnungsgemäß nennen, einen Link zur Creative-Commons-Lizenz beifügen und angeben, ob Änderungen vorgenommen wurden.

Die in diesem Artikel enthaltenen Bilder und sonstiges Drittmaterial unterliegen ebenfalls der genannten Creative-Commons-Lizenz, sofern sich aus der Abbildungslegende nichts anderes ergibt. Sofern das betreffende Material nicht unter der genannten Creative-Commons-Lizenz steht und die betreffende Handlung nicht nach gesetzlichen Vorschriften erlaubt ist, ist für die oben aufgeführten Weiterverwendungen des Materials die Einwilligung des jeweiligen Rechteinhabers einzuholen.

Weitere Details zur Lizenz entnehmen Sie bitte der Lizenzinformation auf http://creativecommons.org/licenses/by/4.0/deed.de.

